# Simultaneous quantification method for 5-FU, uracil, and tegafur using UPLC-MS/MS and clinical application in monitoring UFT/LV combination therapy after hepatectomy

**DOI:** 10.1038/s41598-021-82908-8

**Published:** 2021-02-04

**Authors:** Ken Shiraiwa, Yosuke Suzuki, Hiroki Uchida, Yukio Iwashita, Ryota Tanaka, Motoshi Iwao, Kazuhiro Tada, Teijiro Hirashita, Takashi Masuda, Yuichi Endo, Masafumi Inomata, Hiroki Itoh

**Affiliations:** 1 Department of Clinical Pharmacy, Oita University Hospital, 1-1 Idaigaoka, Hasama-machi, Yufu-shi, Oita, 879-5593 Japan; 2 Department of Gastroenterological and Pediatric Surgery, Faculty of Medicine, Oita University, Yufu-shi, Oita, Japan

**Keywords:** Bioanalytical chemistry, Mass spectrometry, Colorectal cancer, Liver cancer

## Abstract

Combination therapy of tegafur/uracil (UFT) and leucovorin (LV) is widely used to treat colorectal cancers. Although this therapy has a significant therapeutic effect, severe adverse effects occur frequently. Therapeutic drug monitoring (TDM) may help to prevent adverse effects. A useful assay that can quantitate plasma levels of 5-FU, uracil, and tegafur simultaneously for TDM has been desired, but such a method is not currently available. In this study, we aimed to develop a sensitive method for simultaneous quantification of 5-FU, uracil, and tegafur in human plasma using ultra-performance liquid chromatography coupled to tandem mass spectrometry (UPLC-MS/MS). After preparing plasma samples by protein precipitation and liquid extraction, 5-FU, uracil, and tegafur were analyzed by UPLC-MS/MS in negative electrospray ionization mode. Validation was performed according to US Food and Drugs Administration guidance. The calibration curves were linear over concentration ranges of 2–500 ng/mL for 5-FU, 20–5000 ng/mL for uracil, and 200–50,000 ng/mL for tegafur. The corresponding average recovery rates were 79.9, 80.9, and 87.8%. The method provides accuracy within 11.6% and precision below 13.3% for all three analytes. Matrix effects of 5-FU, uracil, and tegafur were higher than 43.5, 84.9, and 100.2%, respectively. This assay was successfully applied to assess the time courses of plasma 5-FU, uracil, and tegafur concentrations in two patients with colorectal liver metastasis who received UFT/LV therapy after hepatectomy. In conclusion, we succeeded to develop a sensitive and robust UPLC-MS/MS method for simultaneous quantification of 5-FU, uracil, and tegafur in human plasma. This method is potentially useful for TDM in patients receiving UFT/LV combination therapy.

## Introduction

Colorectal cancer (CRC) is the leading cause of cancer death in Japan^1^. Surgical excision is the gold standard treatment, and the prognosis of CRC has improved even in patients with locally advanced lesions^2^. However, the 5-year relative survival in patients with distant metastases or stage IV CRC remains only slightly higher than 10%^3^. The liver is the most common metastatic site of CRC, and hepatectomy is considered the most effective treatment for colorectal liver metastasis (CRLM), with reported 5-year post-hepatectomy survival rates of 35–58%^4–7^. However, hepatectomy alone does not necessarily lead to a complete cure, and the recurrence rate after hepatectomy is approximately 70%^8,9^. To reduce the recurrence, postoperative adjuvant chemotherapy for resectable CRLM is clinically essential. Nevertheless, there is a clinical obstacle to overcome: patients who receive postoperative adjuvant chemotherapy tend to develop adverse events. Severe adverse drug reaction is most likely due to impaired drug metabolism after hepatectomy.

Combination therapy of tegafur/uracil (UFT) and leucovorin (LV) is currently considered a tolerable and safe adjuvant chemotherapy regimen for CRLM after hepatectomy^10,11^. UFT contains tegafur and uracil in a 1:4 molar ratio. Tegafur is a prodrug that is converted to 5-fluorouracil (5-FU) in the liver, and uracil and LV enhance the activity of 5-FU based on biochemical modulation. Although this regimen has excellent efficacy, severe adverse effects frequently appear, including myelosuppression, fulminant hepatitis, and diarrhea^12–14^. Moreover, the incidence and grade of these adverse effects are expected to be higher in patients after hepatectomy. Thus, preventing these adverse effects is important clinically.

Therapeutic drug monitoring (TDM) is a useful method that aims to maximize therapeutic effects and minimize the adverse effects of drugs. Among anticancer drugs, methotrexate has been measured in clinical practice for a long time^15^. However, many anticancer drugs including UFT are now available to treat patients with cancers. Reports on the development of quantification methods of anticancer drugs for TDM have been published in recent decades. Several methods for UFT and 5-FU measurements have been reported, but there are problems in terms of run time, complicated procedure, specificity, and sensitivity^16–18^.

Ultra-performance liquid chromatography with tandem mass spectrometry (UPLC-MS/MS) allows sensitive and selective quantification of drugs or endogenous biomarkers in biological matrices^19–22^. UPLC provides higher efficiency with wider ranges of linear velocity, flow rate, and backpressure. Higher backpressure and increased throughput result in superior resolution and sensitivity. UPLC reduces time and cost per sample, thereby improving productivity. In this study, we developed a specific and sensitive UPLC-MS/MS assay for simultaneous quantification of 5-FU, uracil, and tegafur in human plasma. To our knowledge, there is no report to date which describes a method of simultaneous determination of 5-FU, uracil, and tegafur concentrations in human plasma. This method can be applied to TDM in patients receiving UFT/LV combination therapy after hepatectomy.

## Methods

### Materials

The standards of 5-FU and uracil were purchased from Wako Pure Chemical Ind. Ltd. (Osaka, Japan), and the standard of tegafur was purchased from Tokyo Chemical Industry Co. Ltd. (Tokyo, Japan). The stable isotope-labeled internal standards (IS) for 5-FU and tegafur; 5-FU-^13^C,^15^N_2_ and tegafur-^13^C,^15^N_2_, respectively, were synthesized by Toronto Research Chemicals (Toronto, Canada) and that for uracil; uracil-^15^N_2_, was synthesized by Cambridge Isotope Laboratories Inc. (Andover, MA, USA). Ammonium formate used for preparing working solution was purchased from Wako Pure Chemical Ind. Ltd. (Osaka, Japan). All solvents (water, methanol, acetonitrile, ethyl acetate, and isopropyl alcohol) used in chromatography, mass spectrometry, liquid extraction, and sample preparation were of the highest analytical quality (HPLC or LC/MS grade).

### Clinical study and plasma sample collection

The clinical study protocol was approved by the Institutional Review Board (IRB) of Oita University Hospital (approval number: B-15-039) and performed according to the Declaration of Helsinki as amended in Somerset West (1996). After informed consent was obtained from two CRLM patients after undergoing partial hepatectomy, the patients started to take oral tegafur/uracil (UFT; Taiho, Tokyo, Japan) plus leucovorin (UZEL; Taiho, Tokyo, Japan). The patients took UFT (400 mg/day for patient 1 and 500 mg/day for patient 2) and 75 mg/day of UZEL every 8 h on an empty stomach for 28 days. Blood samples were collected before administration (0 min; day 1 and day 8) and after administration (15, 30, 60, 90, 180, and 480 min; day 1 and day 8). After centrifugation, plasma samples were separated and frozen at − 40 °C until assay.

### Stock and working solutions

Stock solutions of 5-FU and 5-FU-^13^C,^15^N_2_ at 100 and 20 µg/mL, respectively, and tegafur and tegafur-^13^C,^15^N_2_ at 1 mg/mL and 20 µg/mL, respectively, in 100% methanol were prepared in 50-mL volumetric flasks, and were dispensed into 2 mL polypropylene tubes. Stock solutions of uracil and uracil-^15^N_2_ at 100 and 20 µg/mL, respectively, in 50% methanol were prepared in 50-mL volumetric flasks and dispensed as for 5-FU and tegafur. Stock solutions for quality control (QC) were prepared separately from standard solutions for the calibration curves. All stock solutions were stored at − 40 °C until use. Calibrating solutions were prepared by diluting the calibration stock solutions in 50% acetonitrile aqueous solution containing 2 mM ammonium formate to the following concentrations: 5-FU (2, 5, 10, 20, 50, 100, 200 and 500 ng/mL), uracil (20, 50, 100, 200, 500, 1000, 2000 and 5000 ng/mL) and tegafur (200, 500, 1000, 2000, 5000, 10,000, 20,000 and 50,000 ng/mL). Lower limit of quantification (LLOQ) and three QC (low, medium and high) solutions were prepared by diluting the QC stock solutions to the following concentration: 5-FU (2, 6, 45 and 400 ng/mL), uracil (20, 60, 450 and 4000 ng/mL) and tegafur (200, 600, 4500 and 40,000 ng/mL).

### Plasma sample preparation

Two hundred µL of blank plasma was added to 100 µL of each calibration or QC solution and vortexed with 100 µL of the internal standard (IS) mixture (5-FU-^13^C,^15^N_2_; 200 ng/mL, uracil-^15^N_2_; 2000 ng/mL, and tegafur-^13^C,^15^N_2_; 5000 ng/mL). The patient sample (200 µL) was also vortexed with 100 µL of the IS mixture and 100 µL of 50% acetonitrile aqueous solution containing 2 mM ammonium formate for volume adjustment. After adding 500 µL of methanol for protein precipitation, all samples were vortexed for 1 min. After centrifugation at 15,000 rpm at 10 °C for 10 min, 750 µL of the supernatant was carefully transferred to a new polypropylene tube and evaporated with nitrogen (N_2_) gas. After evaporation, 1800 µL of ethyl acetate was added and liquid–liquid extraction was performed for 10 min. After centrifugation at 15,000 rpm at 10 °C for 10 min, 1440 µL of the supernatant was transferred to a new polypropylene tube and evaporated with N_2_ gas. The residue was redissolved with 100 µL of 27.5% acetonitrile aqueous solution containing 2 mM ammonium formate.

### Liquid chromatography

The conditions for liquid chromatography were as described previously by Tanaka et al.^21^. All samples were analyzed using the Acquity UPLC I-Class System and a triple-stage quadrupole mass spectrometer (Xevo TQ-D). For chromatographic separation of the analytes, a Waters Acquity HSS T3 column (1.8 µm, 2.1 × 150 mm) preceded by a Waters Acquity HSS T3 Van-Guard pre-column (1.8 µm, 2.1 × 5 mm) was used. The mobile phase used for chromatographic separation consisted of water (solvent A) and acetonitrile (solvent B). The total analysis time was 12 min per injection. The flow rate was 0.2 mL/min, and the gradient was started at 90% solvent A with 10% solvent B (1.0 min). Within 2.0 min, the ratio was changed linearly to 70% solvent A and 30% solvent B. Within the next 2.5 min, the ratio was changed linearly to 10% solvent A and 90% solvent B, and kept stable for 2.0 min. Finally, the gradient returned to the starting conditions for the last 4.5 min. The column temperature was kept at 30 °C during all measurements, while the sample temperature was kept at 5 °C. The injection volume into UPLC-MS/NS was 15 µL.

### Mass spectrometry

The conditions for mass spectrometry were as described previously by Tanaka et al.^21^. Samples were ionized using the following parameters: electrospray voltage was 0.5 kV, cone voltage was 20 V, source temperature was 150 °C, cone gas (N_2_) flow rate was 150 L/hour, desolvation gas (N_2_) flow rate was 1000 L/hour, and desolvation temperature was 500 °C. The mass spectrometer was optimized automatically to 5-FU, uracil, tegafur, and each internal standard, using the MassLynx V4.1 system software package (Waters) and IntelliStart standard optimization procedures (Waters). Multiple reaction monitoring (MRM) analysis was carried out using argon as collision gas for collision-induced dissociation (CID). The transitions from precursor ions to product ions monitored in the negative ion mode for 5-FU, uracil, tegafur, and the corresponding internal standards were *m*/*z* 128.91 → * m*/*z* 41.85 at 28 V, *m*/*z* 110.92 → * m*/*z* 41.84 at 26 V, *m*/*z* 198.98 → * m*/*z* 41.84 at 28 V, *m*/*z* 131.90 → * m*/*z* 43.68 at 28 V, *m*/*z* 112.91 → * m*/*z* 42.82 at 28 V and *m*/*z* 201.99 → * m*/*z* 43.41 at 30 V, respectively, with a dwell time of 52 ms for each transition.

### Validation of the analytical method

Analytical validation was performed according to the guidelines published by the US Food and Drug Administration (FDA)^23^. All analytical parameters concerning validation were determined according to previous reports^19–22^. Three validation batches, each containing eight calibration samples and 24 QC samples at different concentrations (LLOQ, QC A, B, and C; each in sextuplicate), were analyzed. Accuracy was determined as the ratio of mean measured concentration to the nominal concentration. Precision was defined by the ratio of the standard deviation to the mean concentration measured. Accuracy and precision were calculated for each analytical batch (within-batch) and for three validation batches (batch-to-batch). Selectivity was evaluated by comparing chromatograms of blank plasma samples obtained from six healthy volunteers without the addition of the internal standard mixture. The baseline signals at the expected analyte retention times were evaluated for interfering peaks. The recovery rate from plasma was determined by comparing the peak areas obtained from QC samples A, B or C with the respective peak area obtained from blank plasma spiked at the corresponding QC level after extraction (representing 100% of the analyte amount in an identical matrix) in triplicate determination. The potential matrix effect of plasma components affecting the measurement of analytes was calculated by comparing the peak area of blank plasma spiked at QC levels A, B or C after extraction with the respective peak area of matrix-free LC eluent containing the same amount of analyte in triplicate determination. The freeze–thaw stability of analytes was tested using QC samples B and C after undergoing three freeze-and-thaw cycles, and accuracy was calculated. Stability in the autosampler was tested by performing repeated analysis on QC samples B and C after being placed in the autosampler at 5 °C for 24 h. Long-term stability has already been reported^24,25^.

### Incurred sample reanalysis

Incurred sample reanalysis (ISR) was conducted to verify the reliability of the patient sample measurements. All the patient samples were reanalyzed. According to the FDA guidance^23^, the percentage difference of the results between the original measurement and the reanalyzed measurement was determined with the following equation: % Difference = (Reanalyzed – Original)/Mean × 100%.

### Calculations

Calibration curves were obtained using the standard samples and analyte-specific MRM quantifier transitions. Peak area ratios of 5-FU, uracil and tegafur to the corresponding internal standards were calculated, and weighted linear regression (1/x) was carried out for each analytical batch using the TargetLynx V4.1 software package (Waters). Method linearity was validated by performing simple linear regression between the nominal concentrations and back-calculated concentrations of the calibration samples using the corresponding calibration curve.

## Results

### Mass spectrometric and chromatographic characteristics

**Figure 1:**
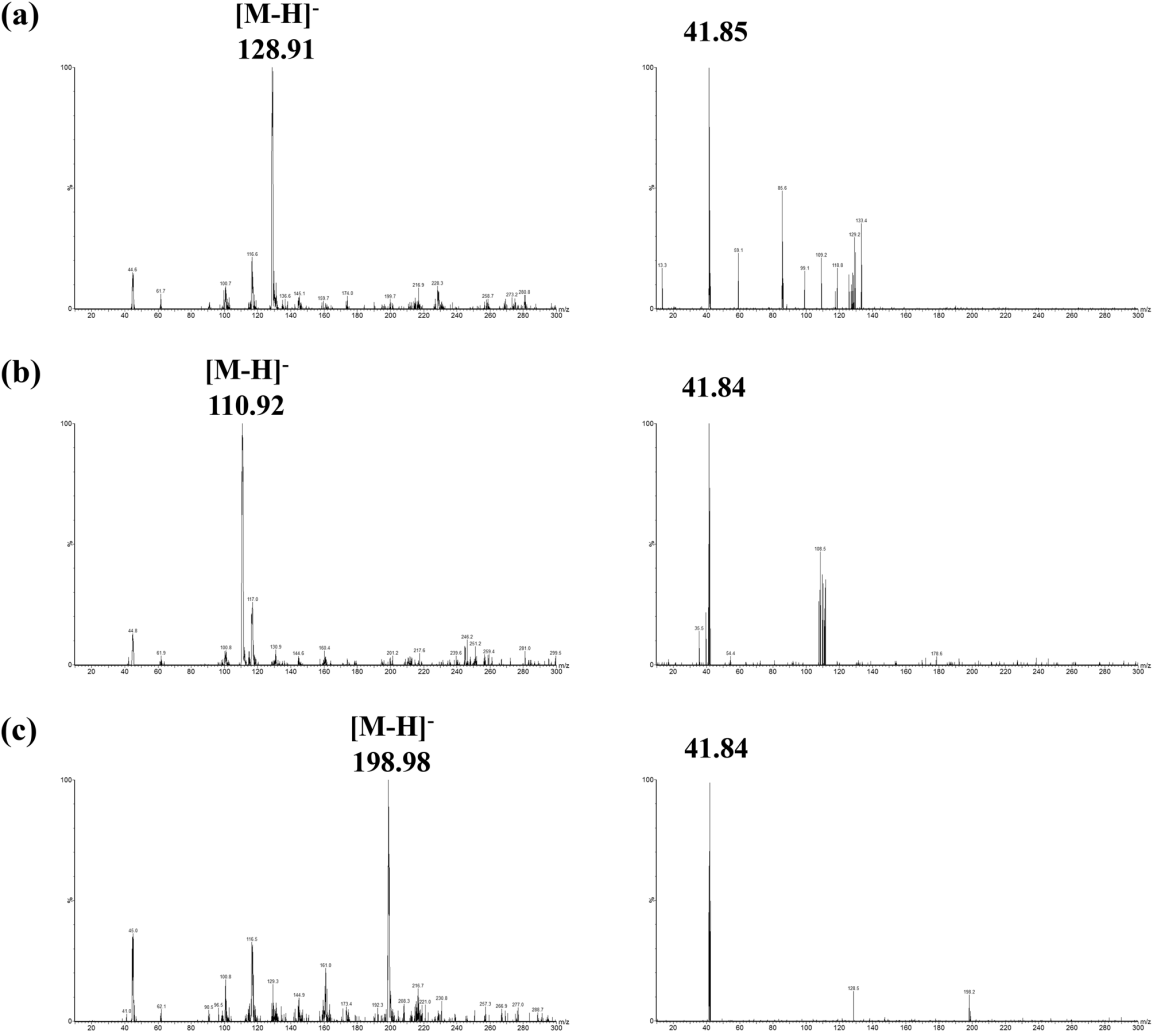
Mass spectra of precursor and product ions. Mass spectra of each precursor ion are shown at the left panels, and mass spectra of each product ion are shown at the right panels: (**a**) 5-FU, (**b**) uracil, and (**c**) tegafur. Strong [M-H]^-^ signals for these three compounds were detected using negative electrospray ionization. To select identical fragment ions for these three compounds and their ISs, sensitive detection by MRM analysis was carried out.

**Figure 2:**
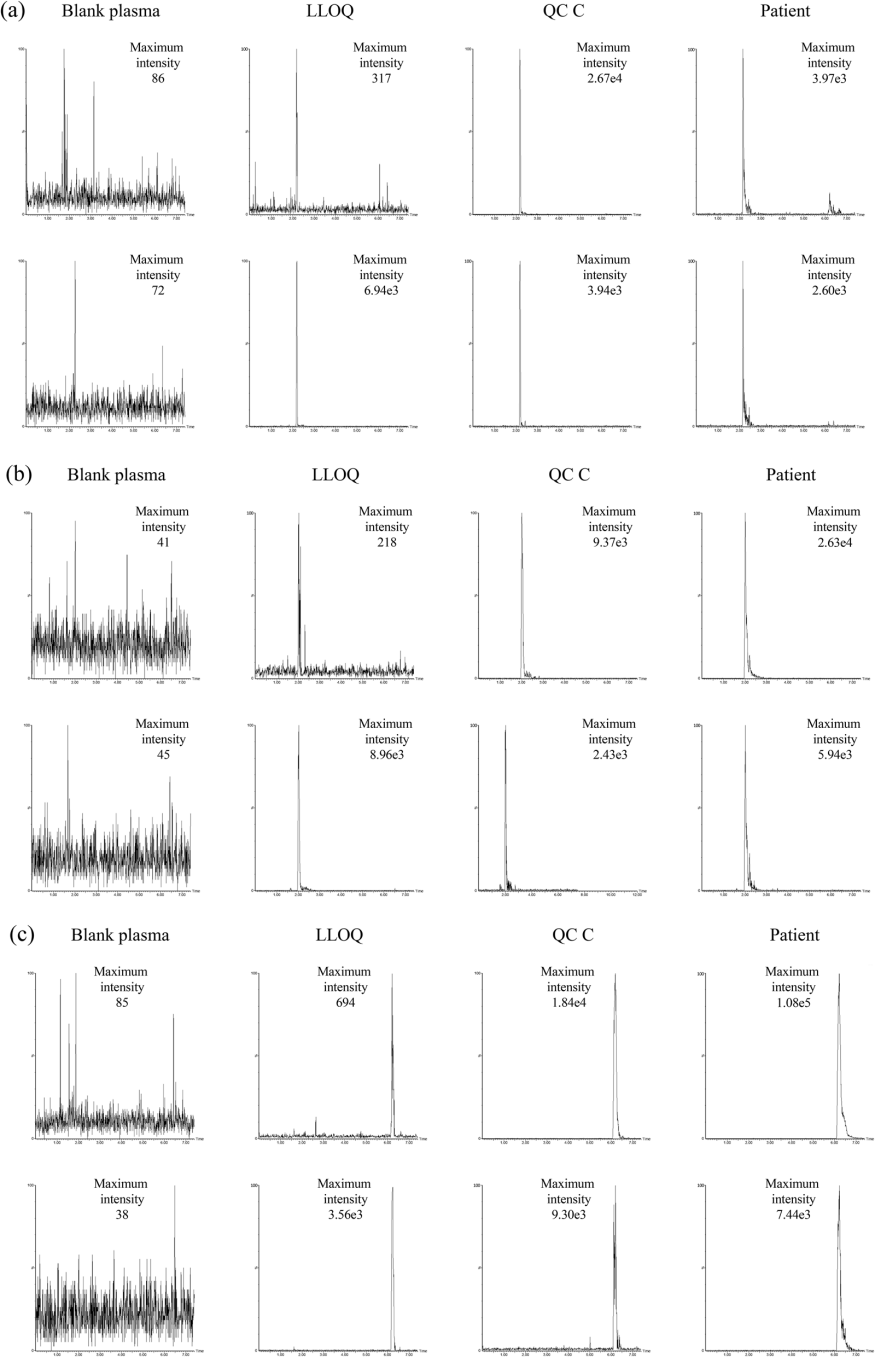
Representative chromatograms of standards and internal standards. Representative chromatograms of standards and internal standards are shown at upper panels and lower panels, respectively: (**a**) 5-FU, (**b**) uracil, and (**c**) tegafur. No interfering peaks due to plasma matrix were observed in plasma samples obtained from healthy volunteers. A good peak shape was also obtained in LLOQ. LLOQ, lower limit of quantification; QC, quality control.

Figure 1 shows the precursor ion and product ion mass spectra of 5-FU, uracil, and tegafur. Intense [M-H]- signals for these three compounds were detected using negative electrospray ionization. To select identical fragment ions for these three compounds and their ISs, sensitive detection by MRM analysis was carried out. The similar fragment ions of 5-FU and IS of 5-FU were *m*/*z* 41.85 and 43.68, respectively, which differed by approximately *m*/*z* 2 due to isotopic labeling. The identical fragment ions of uracil and IS of uracil were *m*/*z* 41.84 and 42.82, respectively, with a difference of approximately *m*/*z* 1. The similar fragment ions of tegafur and IS of tegafur were *m*/*z* 41.84 and 43.41, respectively, and differed by approximately *m*/*z* 2. The chromatographic conditions were optimized for rapid and efficient separation of these three compounds and the ISs from other substances in plasma. Figure 2 shows the chromatographic characteristics of these three compounds and the corresponding ISs in blank plasma, LLOQ, high QC, and plasma sample of a patient undergoing UFT/LV combination therapy. Under the optimized chromatographic conditions, the retention time was 2.00 min for 5-FU and IS of 5-FU, 2.17 min for uracil and IS of uracil, and 6.08 min for tegafur and IS of tegafur.

### Validation results

As shown in Fig. 2, no interfering peaks due to plasma matrix were observed in plasma samples obtained from six different individuals, demonstrating acceptable selectivity of this method. A good peak shape was also obtained at the LLOQ of each compound. Full validation was performed according to the US Food and Drug Administration (FDA) guidance. For the calibration curves, the correlation coefficient (r^2^) was ≥ 0.9952 for 5-FU (2 to 500 ng/mL), ≥ 0.9937 for uracil (20 to 5000 ng/mL), and ≥ 0.9916 for tegafur (200 to 50,000 ng/mL). Linearity of each calibration curve was confirmed with eight calibrating solutions. For assessment of method linearity, the regression coefficient (r^2^) was 0.9963 for 5-FU, 0.9946 for uracil, and 0.9941 for tegafur, which validated method linearity for the three analytes. Within-batch and batch-to-batch accuracy and precision data are presented in Table 1. Within-batch accuracy for 3 QCs was between − 1.51 and 9.50% for 5-FU, between − 2.54 and 2.12% for uracil, and between − 9.09 and 11.60% for tegafur. Within-batch accuracy at LLOQ varied between 3.30 and 10.50% for 5-FU, − 8.49 and 4.45% for uracil, and − 2.58 and 6.56% for tegafur. Within-batch precision for 3 QCs was between 2.88 and 12.80% for 5-FU, between 4.34 and 13.30% for uracil, and between 1.75 and 11.60% for tegafur. Within-batch precision at LLOQ varied between 9.32 and 15.18% for 5-FU, between 11.80 and 15.63% for uracil, and between 3.40 and 10.80% for tegafur. Batch-to-batch accuracy for 3 QCs was between 3.65 and 4.80% for 5-FU, between − 1.37 and 0.82% for uracil, and between 0.48 and 3.07% for tegafur. Batch-to-batch precision for 3 QCs was less than 9.27% for 5-FU, less than 8.85% for uracil, and less than 9.72% for tegafur. The extraction recovery rate and matrix effect were evaluated by triplicate determination of 3 QCs for each compound. The average recovery rates (mean ± SD) of 5-FU, uracil, and tegafur were 79.9 ± 11.2, 80.9 ± 5.9, and 87.8 ± 10.6%, respectively. Matrix effects of 5-FU, uracil, and tegafur ranged from 43.5 to 69.7, 84.9 to 102.2, and 100.2 to 119.8%, respectively. There was no difference in matrix effect among all QC levels. The freeze-and-thaw stability was tested within the validation process using three freeze-and-thaw cycles at QC B and C levels. No significant changes in measured concentrations were observed (accuracy ranged between − 0.2 and 9.8% for 5-FU, between − 5.7 and − 3.6% for uracil, and between 2.2% and 20.7% for tegafur). Stability in the autosampler at 5 °C for 24 h was evaluated based on the accuracy for QC B and C samples. The accuracy for 5-FU, uracil, and tegafur measurements ranged from − 5.7 to − 2.6%, − 8.2 to − 4.3% and − 10.5 to − 4.6%, respectively. Since the range of accuracy fell within 15%, autosampler stability was acceptable.

**Table 1:** Results of validation of the novel UPLC-MS/MS method developed in the present study for simultaneous quantification of 5-FU, uracil, and tegafur concentrations in human plasma.

		Nominal 5-FU concentration (ng/mL)				Nominal uracil concentration (ng/mL)				Nominal tegafur concentration (ng/mL)			
		LLOQ	QC A	QC B	QC C	LLOQ	QC A	QC B	QC C	LLOQ	QC A	QC B	QC C
		2	6	45	400	20	60	450	4000	200	600	4500	40,000
**Within-batch**													
1	Mean (ng/mL)	2.07	6.57	46.90	431.93	18.30	59.56	444.80	4084.68	210.12	650.96	4788.93	44,641.84
	Accuracy (%)	3.30	9.50	4.22	7.98	− 8.49	− 0.73	− 1.16	2.12	5.06	8.49	6.42	11.60
	Precision (%CV)	10.50	7.25	3.81	5.62	11.80	7.67	4.34	4.52	3.46	1.75	3.06	2.89
2	Mean (ng/mL)	2.10	5.92	47.82	426.02	20.89	59.13	447.88	4079.61	194.83	602.53	4671.74	41,249.38
	Accuracy (%)	4.75	− 1.38	6.27	6.51	4.45	− 1.45	− 0.47	1.99	− 2.58	0.42	3.82	3.12
	Precision (%CV)	15.18	12.78	6.60	5.07	12.46	13.33	6.58	9.04	10.64	11.65	9.92	7.71
3	Mean (ng/mL)	2.21	6.18	46.83	393.96	19.49	61.03	438.57	3934.54	213.12	545.44	4104.53	38,935.85
	Accuracy (%)	10.50	3.00	4.07	− 1.51	− 2.56	1.72	− 2.54	− 1.64	6.56	− 9.09	− 8.79	− 2.66
	Precision (%CV)	9.32	6.91	6.49	2.88	15.63	7.92	6.48	5.81	10.80	4.36	6.23	8.56
**Batch-to-batch**													
	Mean (ng/mL)	2.12	6.22	47.16	415.17	19.38	59.94	443.81	4032.94	205.61	602.87	4521.73	41,228.62
	Accuracy (%)	5.96	3.65	4.80	3.79	− 3.10	− 0.10	− 1.37	0.82	2.80	0.48	0.48	3.07
	Precision (%CV)	11.14	9.27	5.53	6.10	13.40	8.85	5.46	6.58	9.13	9.72	9.43	8.68

*LLOQ* lower limit of quantification, *QC* quality control, *CV* coefficient of variation.

### Plasma concentrations of patients undergoing UFT/LV combination therapy

**Figure 3:**
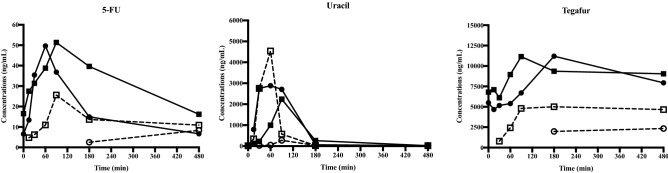
Time courses of plasma concentrations for 5-FU, uracil, and tegafur. Time courses of plasma concentrations in two patients are shown at Fig. 3: patient 1 at day 1 (○) and day 8 (●), patient 2 at day 1 (□) and day 8 (■). Right, central, and left figures are shown for 5-FU, uracil, and tegafur, respectively. At day 1, several data points of 5-FU, uracil, and tegafur were lower than LLOQ because of the absorption lag. However, all measured concentrations of 5-FU, uracil, and tegafur at day 8 were within the calibration ranges.

The time courses of plasma concentrations in two patients receiving UFT/LV combination therapy measured by the UPLC-MS/MS method developed in this study are shown in Fig. 3. The two patients underwent partial hepatectomy for CRLM before starting UFT/LV chemotherapy. One patient received UFT three times a day at a dose of 400 mg/day (200-100-100), and the other received UFT three times a day at a dose of 500 mg/day (200-200-100). At day 1, several data points of 5-FU, uracil, and tegafur were lower than LLOQ because of the absorption lag. At day 8, however, all measured concentrations of 5-FU, uracil, and tegafur were within the calibration ranges. ISR was conducted to verify the patient sample concentrations' reliability by reanalyzing all the patient specimens used in this study. Results of ISR showed that 71.4% for 5-FU, 78.6% for uracil, and 92.9% for tegafur had % difference within ± 20% when calculated with the ISR equation. According to the FDA acceptance criterion, at least 67% of the ISR samples should have % difference within ± 20%. Therefore, the ISR results met the FDA acceptance criteria for all three compounds. These results indicate that the novel method is potentially useful for TDM in patients receiving UFT/LV combination therapy.

## Discussion

To the best of our knowledge, it is the first report of a method for simultaneous quantification of 5-FU, uracil, and tegafur. A method for simultaneous determination of tegafur and 5-FU was reported previously^17^. However, this method cannot be used to measure uracil together with 5-FU and tegafur simultaneously. Uracil inhibits the degradation of 5-FU by dihydropyrimidine dehydrogenase (DPD) in a competitive manner. High uracil levels are associated with higher toxicity as well as efficacy. To maintain an optimal balance between effectiveness and toxicity, tegafur and uracil are combined in a molar ratio of 1:4 in the preparation of UFT^26^. Besides, it is known that hepatectomy causes a reduction in DPD activity, leading to elevation of not only plasma 5-FU but also uracil level^27,28^. In other words, it is critically important to monitor these three compounds to accurately assess the therapeutic and adverse effects of this combination therapy. For these reasons, we developed a novel method to quantify 5-FU, uracil, and tegafur simultaneously in this study.

We used a combination of protein precipitation and liquid extraction for sample preparation. Indeed, protein precipitation is a speedy and straightforward method for sample preparation. However, protein precipitation alone is insufficient to remove matrices that interfere with ionization. Moreover, considering routine use of the MS system, using a combination of sample preparation procedures significantly reduces the wear of the MS system. A combination of protein precipitation and liquid extraction is expected to improve the recovery rate and reduce the matrix effect. Furthermore, simultaneous and more selective detection of 5-FU and uracil was achieved by using tandem mass spectrometry instead of a UV detector system. However, in order to quantify 5-FU, uracil, and tegafur simultaneously, the conditions for sample preparation, MS system, and LC system in our method were set in favor of tegafur. As a result, the recovery rates of the highly polar compounds; 5-FU and uracil, were lower than that of tegafur. In a previously reported combined LC-UV and LC–MS/MS method for simultaneous determination of 5-FU and tegafur, the calibration range of 5-FU was 8–200 ng/mL, and that of tegafur was 800–20,000 ng/mL^17^. Despite using conditions favorable for measuring tegafur, our method has a more extensive calibration range and superior LLOQ for 5-FU compared to the previous method.

Severe adverse effects tend to occur during adjuvant chemotherapy given after liver resection compared to without liver resection. We previously reported that major hepatectomy enhanced the toxicity of 5-FU in a rat model^27^. The changes in drug metabolism after hepatectomy observed in the rat model probably also apply to humans. UFT/LV combination therapy is considered more tolerable and safer than oxaliplatin or irinotecan-based regimen. Although we consider UFT/LV the most recommended regimen for CRLM patients after hepatectomy, the impact of hepatectomy on the pharmacokinetics of UFT/LV remains unclear. Therefore, the optimal dose of UFT in this regimen cannot be determined. To provide more reliable therapy, TDM is a useful approach.

After our novel method was developed and validated, we examined the clinical application of this method to TDM for patients receiving UFT/LV therapy. Although several data points of each compound at day 1 were lower than LLOQ, all data points at day 8, when steady state was assumed to have reached, were within the calibration ranges. These results demonstrate that the method is clinically applicable for TDM of patients undergoing UFT/LV therapy.

Currently, the department of gastroenterological and pediatric surgery and the department of clinical pharmacy in Oita University Hospital are jointly conducting phase I clinical study (UMIN 000,021,146) designed to elucidate the effect of major hepatectomy, as reflected by the efficacy and toxicity of UFT/LV combination therapy. In the future, we shall evaluate the impact of major hepatectomy on drug metabolism in patients on UFT/LV combination therapy.

## Conclusion

We have succeeded to develop a method for simultaneous quantification of 5-FU, uracil, and tegafur using UPLC-MS/MS. Subsequently, we verified the clinical application of this method. Our novel method is potentially useful for TDM in CRLM patients receiving UFT/LV therapy after hepatectomy.
